# Expression of the neurodevelopmental disease susceptibility genes *CADM1* and *CADM2* in the early human fetal forebrain

**DOI:** 10.1111/joa.70248

**Published:** 2026-11-10

**Authors:** Natalie Wucherer, Maznah Alhesain, Amn Ashhad, Marc Woodbury‐Smith, Gavin J. Clowry

**Affiliations:** ^1^ Centre for Neuroscience Newcastle University Biosciences Institute Newcastle Upon Tyne UK; ^2^ Present address: Department of Anatomy and Histology, College of Medicine Qassim University Buraydah Saudi Arabia; ^3^ Present address: Division of Developmental Disabilities, Department of Psychiatry Queen's University Kingston Canada

**Keywords:** cell‐adhesion molecules, cerebral cortex, corpus callosum, development, thalamocortical, thalamus

## Abstract

CADM1 and CADM2 are cell‐adhesion molecules with likely roles in cell migration, axon guidance and synapse formation. Both are considered potential susceptibility genes for various neurodevelopmental disorders. However, their expression patterns in early human brain development have not been explored. Here we employed transcriptomics, RNAScope in situ hybridisation (ISH) and immunofluorescence (IF) to determine the time course and location of expression in the human cerebral cortex and thalamus between 7.5–17 postconceptional weeks (PCW). In the cerebral cortex, tissue RNAseq revealed both to be highly expressed throughout this period. *CADM1* exhibited very high expression which declined slightly with age. *CADM2* exhibited high expression which increased over time. Single‐cell RNA‐seq and ISH revealed that both genes were co‐expressed in most cells; however, IF revealed different patterns of cellular localisation. At 8 PCW, expression for both CADMs was similar throughout the cortex and predominantly located in the cytoplasm/cell surface in the cortical plate (CP) but with stronger expression of CADM1 in the subventricular zone. However, from 10 PCW onwards, the strongest immunoreactivity for CADM1 was in the cell nucleus with weak cytoplasmic localisation, whereas CADM2 was expressed on the cell surface and increasingly in axons with age. By 17 PCW, CADM2 exhibited strong immunoreactivity in the thalamocortical and callosal corticocortical axons but was weakly expressed in corticofugal pathways. In the thalamus, CADM2 expression in posterior and lateral projection neurons contributing to thalamocortical pathways was confirmed. CADM1 and CADM2 show differential expression in the thalamus and associated nuclei, suggesting a role in their segregation; CADM1 favouring anterior and medial thalamic nuclei and the ventromedial subthalamus, CADM2 the lateral habenula, lateral and posterior thalamus and dorsolateral subthalamus. CADM1 and CADM2 may play important and varied roles in early fetal human forebrain development, guiding cell migration and aggregation, and axon guidance and fasciculation.

## Graphical abstract

CADM1 and CADM2 are related cell‐adhesion molecules that show differential patterns of expression in early human fetal forebrain. Although often co‐expressed in neurons, CADM2 protein exclusively and selectively localises to axons, whereas CADM1 is predominantly expressed in the nucleus, posing questions as to what their developmental roles might be.

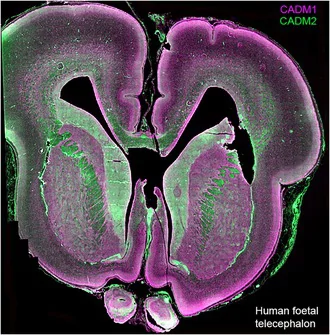

## 1 INTRODUCTION

CADM1 (cell‐adhesion molecule 1 also known as SYNCAM1) and CADM2 (cell‐adhesion molecule 2 also known as SYNCAM2) are both glycoproteins and members of the immunoglobulin superfamily (Chang et al., 2010). The gene for *CADM1* is located at chromosome 11q23.3, and for *CADM2* it is located at 3p12.1 in humans (Chang et al., 2010; Fujita et al., 2005). Both have been identified as susceptibility genes for various neurodevelopmental disorders.

Mutations in CADM2 are associated, primarily through genome‐wide association studies, with risk‐taking behaviour (Truvé et al., 2020), increased substance use, increased body mass index and paediatric‐onset type 2 diabetes (Greenbaum et al., 2019). It is speculated that the role CADM2 plays in synaptic connectivity could contribute to reward‐learning and associative learning, resulting in association with risky behaviour (Pasman et al., 2022). It has also been suggested as a candidate susceptibility gene for Autism Spectrum Disorder (ASD; Casey et al., 2012), Attention Deficit Hyperactivity Disorder (ADHD; Koller et al., 2024) and developmental dyslexia (Doust et al., 2022). CADM2 has few binding partners (Shirogane et al., 2021; Szklarczyk et al., 2022) but is known to interact with CADM1. In chicken embryos, it has been shown that floor plate‐expressed CADM2 guides axons across the midline and then rostrally by interacting with growth cone‐localised CADM1/CADM2 complexes (Niederkofler et al., 2010).

*CADM1* mutations have also been linked to ADHD (Jin et al., 2019) and ASD (Zhiling et al., 2008). The likely roles of CADM1 and CADM2 in brain development include effects upon cell migration, axonal growth and guidance and synapse formation during early cortical development. Therefore, we sought to establish to what extent CADM1 and CADM2 are expressed in fetal human forebrain between 7.5 and 17 postconceptional weeks (PCW), by which cell types and in which locations, in order to generate hypotheses as to how CADM1 and CADM2 might be involved in developmental processes at this stage.

CADM1 has cell‐adhesion properties that promote axonal growth, neuronal migration, pathfinding and synaptic formation in the developing nervous system and in the formation of neural networks in mice (Fujita et al., 2005) and has been shown to restrict the number of active filopodia during axonal growth by localising to growth cones in developing rat neurons (Stagi et al., 2010). CADM1 has been localised to both the pre‐ and post‐synaptic sides of the synaptic cleft and interacts with scaffold proteins such as MUPP1 (present at tight junctions) in the mouse brain (Fujita et al., 2012) and CASK (found at synapses) in HEK293 cells and rat brain (Biederer et al., 2002). CADM1 has been found to have Ca^2+^‐independent homophilic cell‐adhesion properties at both pre‐ and post‐synaptic sites (Biederer et al., 2002). *Cadm1* mutations in mouse‐derived cell cultures cause alterations in neurons, including shorter dendrites, impaired synaptogenesis and disruption in protein distribution, with abnormal proteins accumulating in the endoplasmic reticulum (ER). This aggregation has been shown to cause ER stress and neuronal death, potentially leading to aberrant neurodevelopment and related pathogenesis (Fujita et al., 2010).

CADM2 engages with nectin‐like proteins in homo‐ and heterophilic interactions to promote cell aggregation and regulation of trans‐synaptic adhesion in the central nervous system (CNS). Expression of only the cytoplasmic tail of CADM2 within neurons results in an inhibition of synapse assembly, while the expression of the full‐length CADM2 protein induces synapse assembly when expressed in non‐neuronal cells (Truvé et al., 2020). This protein product is primarily found in neurons and localised to synapses of the adult human CNS, particularly in the hippocampus and frontal and anterior cingulate cortex (Thomas et al., 2008).

Neurodevelopmental diseases may arise from loss, gain or aberrant function of key proteins in very early cortical development, altering the trajectory of circuit formation (Molnár et al., 2019). The aim of this study was to shed light on how mutations in *CADM1* and *CADM2* might lead to the maldevelopment of cortical circuitry.

## 2 METHODS AND MATERIALS

### 2.1 Transcriptomics (tissue and single cell)

Tissue RNA‐seq data were obtained from the HDBR expression database (https://www.hdbr.org/expression) which utilised the dataset deposited at www.ebi.ac.uk/arrayexpress/experiments/E‐MTAB‐4840 taken from 138 discrete human cortical tissue samples from anterior, posterior and temporal regions of the cortex between the ages of 7.5 and 17 PCW, expressed in Reads Per Kilobase per Million mapped reads (RPKM; Lindsay et al., 2016). These data were analysed using Prism software, where Rout's outlier test was applied.

Thalamic tissue RNAseq data were obtained from the BrainSpan Developmental Transcriptome (www.brainspan.org). These data were taken from mice included between 8 and 17 PCW from the dorsal thalamus and mediodorsal nucleus. These data were analysed using Prism after log2 transformation was removed from the data.

Single Cell Transcriptomic data at 16 PCW was obtained from NEMO Analytics (nemoanalytics.org) and their Single Cell Workbench was utilised (Orvis et al., 2021) on default settings with five dimensions to generate UMAP plots for the thalamus (Developmental timepoint and Brain region GW18_thalamus from Human—Single Cell 10X RNAseq—Kriegstein), visual cortex (Developmental timepoint and Brain region GW18_V1 from Human—Single Cell 10X RNAseq—Kriegstein) and prefrontal cortex (scRNAseq of human prefrontal cortex at GW18). Genes whose expression was utilised as markers for identifying UMAP clusters can be found in Table 1.

**TABLE 1:** Marker genes used for generating cell cluster maps in Figures 1 and 4.

Cell type	Gene	References
Immature neuron (migrating)	*DCX*	Ayanlaja et al. (2017)
Developing neuron (axon extending)	*GAP43*	Denny (2006)
Thalamic glutamatergic neurons	*SLC17A6*	Kim et al. (2023)
Cortical glutamatergic neurons	*SLC17A7*	Liguz‐Lecznar and Skangiel‐Kramska (2007)
Cortical glutamatergic L2/3	*CUX2*	Cubelos et al. (2010)
Cortical glutamatergic L4	*RORB*	Clark et al. (2020)
Cortical glutamatergic L5	*CRYM*	Hommyo et al. (2018)
Cortical glutamatergic L6	*TLE4*	Zeng et al. (2012)
GABAergic neurons	*GAD1*	Grone and Maruska (2016)
Oligodendrocyte precursor cells (OPC)	*PDGFRA*	Zhu et al. (2014)
Radial glia	*HES1*	Dhanesh et al. (2016)
Ventricular radial glia	*CRYAB*	Nowakowski et al. (2016)
Outer radial glia	*HOPX*	Nowakowski et al. (2016)
Microglia	*CCL3*	Chui and Dorovini‐Zis (2010)
Actively dividing progenitors	*MKI67*	Sobecki et al. (2016)
Red blood cells	*HBA1*	Mussolino and Strouboulis (2021)
Pericytes and endothelial cells	*BGN*	Hu et al. (2016)

### 2.2 Human tissue

Human fetal tissue from terminated pregnancies was obtained from the joint MRC/Wellcome Trust‐funded Human Developmental Biology Resource (HDBR; www.hdbr.org; Gerrelli et al., 2015). All tissue was collected with appropriate maternal consent and approval from the Newcastle and North Tyneside NHS Health Authority Joint Ethics Committee. This study used fetal samples aged between 8 and 17 PCW. Samples were dissected, fixed in buffered 4% paraformaldehyde solution and embedded in paraffin before being sectioned according to standard protocols applied by the HDBR staff (www.hdbr.org/factsheets).

### 2.3 Multiple immunofluorescence

Immunofluorescence staining was conducted according to previously described protocols (Alzu'bi et al., 2022) with various primary antibodies (Table 2). This protocol used horseradish peroxidase (HRP) conjugated secondary antibodies (Vector Lab ImmPRESS™ HRP IgG [Peroxidase] Polymer Detection Kits, Goat MP‐7451‐15 and Horse MP‐7402; B2B Scientific, Peterborough, UK) developed with Tyramide System Amplification using Opal TSA 520 (OP‐001001; Akoya Biosciences, Marlborough, MA, USA) or 570 (OP‐001003, Akoya Biosciences) fluorescent dyes. Sections were mounted using ProLong Gold antifade reagent with DAPi (Thermo Fisher, Horsham, UK).

**TABLE 2:** Primary antibodies and RNAscope probes employed.

Antibody target	Species	Experimental dilution	Company	RRID number
CADM2	Rabbit polyclonal	1/1000	Invitrogen Horsham, UK	AB_2550758
GAP 43	Mouse monoclonal	1/500	Santa Cruz Biotech Heidelberg, Germany	AB_11140128
Beta‐3 Tubulin	Mouse monoclonal	1/500	Abcam, Cambridge, UK	AB_306045
CADM1	Rabbit polyclonal	1/1000	Invitrogen	AB_2541696
PAX6	Rabbit polyclonal	1/1000	Abcam	AB_2750924
NR2F2	Mouse monoclonal	1/500	Perseus Proteomics	AB_2314222
FOXP2	Mouse monoclonal	1/50	Santa Cruz Biotech	AB_2721204
ROBO1	Rabbit polyclonal	1/500	Abcam	AB_449561
SLIT2	Rabbit polyclonal	1/1000	Proteintech, Manchester, UK	AB_10805766

ACD probe	Cat No.	Gene ID	Target region and probe size	Accession number
*Hs‐CADM1*	470071	23705	243–1254 20zz	NM_014333.3
*Hs‐CADM2*	1139141‐C2	253559	1682–3622 20zz	NM_001167674.2

### 2.4 RNAscope in situ hybridisation coupled with immunofluorescence

RNAscope in situ hybridisation (ISH) with or without coupling with protein immunofluorescence was conducted following the protocol described in Alzu'bi et al. (2022). RNAscope probes for *CADM1* and *CADM2* from ACD Bio Techne (Abingdon, UK; details in Table 2) were utilised along with antibodies listed in Table 2.

### 2.5 Imaging

Fluorescent images were obtained with a Zeiss Axioimager Z2. Processing of images, which involved adjustment of brightness, colour balance and sharpness, was achieved using Zeiss Zen Imaging software.

## 3 RESULTS

### 3.1 The cerebral cortex between 7.5 and 17 PCW

Whole tissue RNA‐seq showed that *CADM1* expression was very high but decreased moderately but significantly within the cortex between 7.5 and 17 PCW, with the mean expression level (126.2 RPKM) placing it within the top 10% of all protein‐coding genes during this developmental period. *CADM2* expression increased moderately but significantly between 7.5 and 17 PCW and was not as highly expressed, although its mean expression (47.6 RPKM) still placed it in the top quartile of protein‐coding genes (Harkin et al., 2017; Figure 1a). *CADM1* and *CADM2* expression were negatively and significantly correlated (Figure 1b). Examining expression by cortical region showed that *CADM1 expression* increased in a statistically significant gradient from frontal to temporal lobe, with *CADM2* expression trending in the opposite direction but not reaching statistical significance (Figure 1c).

**FIGURE 1:**
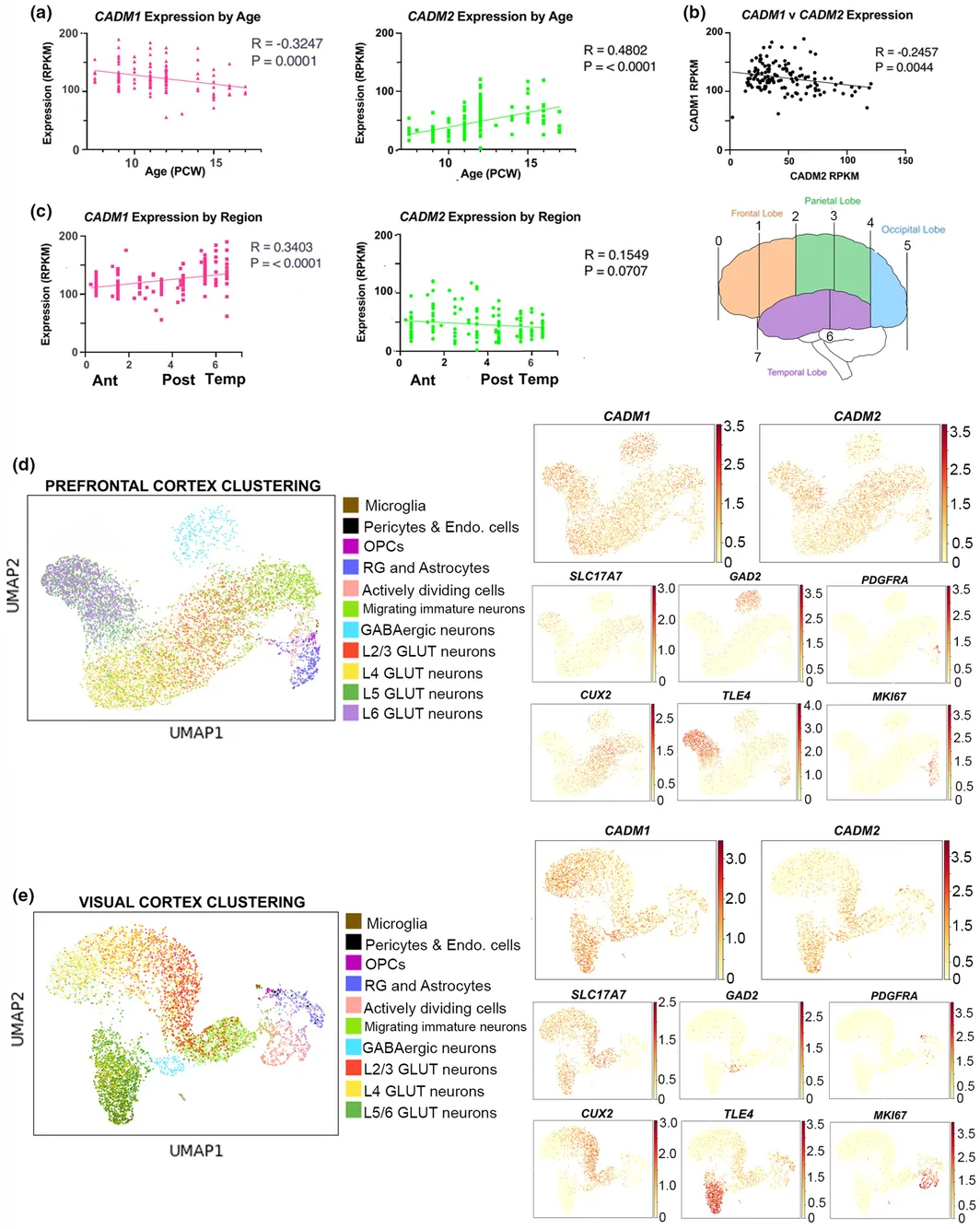
Cortical transcriptomics. Cortical tissue RNA‐seq presented as RPKM (from www.ebi.ac.uk/arrayexpress/experiments/E‐MTAB‐4840) for CADM1 and CADM2 by age of sample in postconceptional weeks (PCW) (a), CADM1 plotted against CADM2 by sample (b), CADM1 and CADM2 by cortical location of sample with reference diagram (c). Single‐cell RNA‐seq data at 16 PCW (from nemoanalytics.org) for the prefrontal cortex (d) and visual cortex (e), including specification of cell clusters by specific markers and showing individual gene expression UMAPS for CADM1 and CADM2 and key example marker genes (see Table 1). Endo., endothelial; RG, radial glia; GLUT, glutamatergic.

Single‐cell RNA‐seq analysis of the prefrontal (Figure 1d) and visual (Figure 1e) cortex at 16 PCW gave similar results and showed that *CADM1* and *CADM2* have broad cell‐type expression, although *CADM1* expression favoured GABAergic neurons while *CADM2* favoured glutamatergic neurons and putative oligodendrocyte precursor cells (OPCs). *CADM1* was also found to favour cells expressing putative cortical layer 4, 5 and 6 neuronal markers, whereas *CADM2* favoured cells expressing putative layer 2/3, 5 and 6 markers. It is important to note that overlap in UMAP space between the layer 5 marker CRYM‐expressing cells and layer 6 marker TLE4‐expressing cells made it difficult to identify if *CADM2* expression favoured layer 5, 6 or both. At this stage of development, only layer 4, 5 and 6 neurons have settled in the cortical plate (CP; Akula et al., 2023; Bayatti et al., 2008), suggesting that identified layer 2/3 neurons must be relatively immature and still migratory. Both *CADM1* and *CADM2* showed weak expression amongst both quiescent and actively dividing progenitor cell populations.

Using histological approaches, we confirmed CADM1 and CADM2 expression, both mRNA and protein, in the cerebral cortex at eight PCW. CADM2 protein was strongly expressed throughout the cortical plate (CP), where post‐mitotic neurons are located, but was shown here in the dorsofrontal cortex, confirmed by demonstrating colocalization of CADM2 with β‐Tubulin immunoreactivity (Figure 2a) and was weakly expressed in the progenitor zones, but was not localised to GAP43+ axons in the intermediate zone (IZ; Figure 2b). Both CADM1 and CADM2 were expressed within the CP, subventricular zone (SVZ) and ventricular zone (VZ) and largely colocalised to the same cell bodies. CADM1, like CADM2, was not expressed in the intermediate zone (IZ), a predominantly axonal compartment (Figure 2c). CADM1 immunoreactivity was found to colocalize with PAX6, a nuclear marker for neuroprogenitor cells in the human VZ and SVZ (Bayatti et al., 2008; Figure 2d). The *CADM1* mRNA, demonstrated by RNAScope ISH, was co‐expressed with CADM1 protein across all cortical layers, including the pre‐subplate (pSP) and marginal zone (MZ), with the strongest expression and colocalization in the CP (Figure 1e). *CADM2* RNA also colocalised with its protein in the CP but, unlike for *CADM1*, the mRNA was only found to be strongly expressed within the MZ, CP and pSP but not in the progenitor zones (Figure 2f). Colocalization in CP cells was also observed between *CADM1* and *CADM2* mRNA (Figure 2g).

**FIGURE 2:**
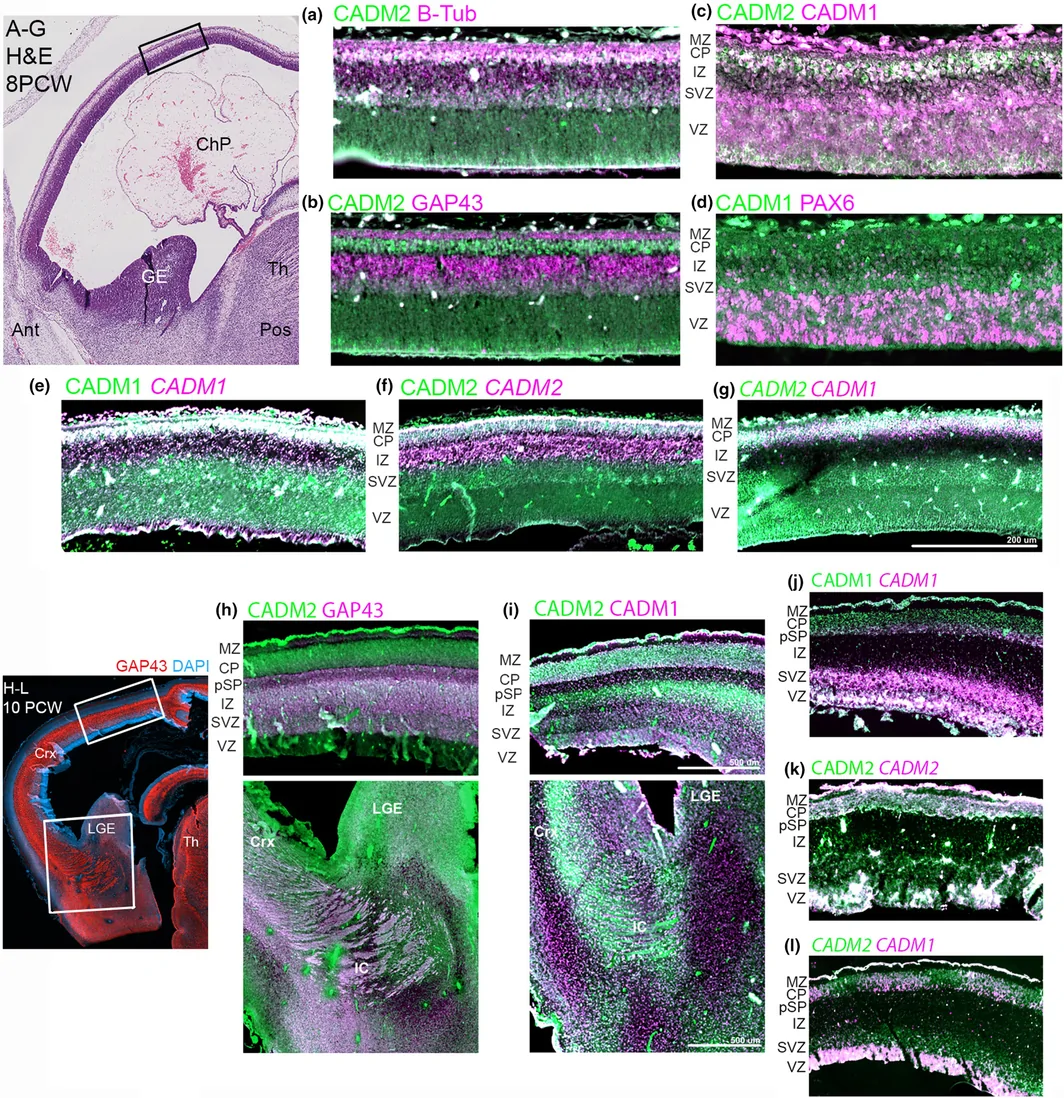
Cortical expression of CADM1 and CADM2 between 8 and 10 PCW. Eight PCW: Lower power inset from section stained with haemotoxylin and eosin (H&E) with box showing approximate location of higher power images A‐G. Dual Immunofluorescence for CADM2 (green) and β‐Tubulin (red; a); CADM2 and GAP43 in the cortical wall (b); CADM2 and CADM1 (c) and CADM1 and PAX6 (d), all at 8 PCW. RNAscope ISH + Protein Immunofluorescence for *CADM1* mRNA (magenta) and protein (green; e), CADM2 mRNA and protein (f). RNAscope multichannel of *CADM1* mRNA (magenta) and *CADM2* mRNA (green, g). Note that both are highly expressed in the cortical plate (CP) in β‐tubulin+ post‐mitotic neurons and largely co‐expressed in the same cells (colocalization of magenta and green appears white). Expression is also observed in the PAX6+ proliferative zones, but there is no co‐expression in GAP43+ axons in the presubplate (pSP) and intermediate zone (IZ). Ten PCW: Lower‐power inset from a section immunostained for GAP43 and counterstained with DAPI, with boxes showing the approximate location of higher‐power images H‐L. Dual immunofluorescence of the cortex and internal capsule (IC) stained for CADM2 (green) and GAP43 (magenta; h) and CADM2 and CADM1 (i). RNAscope + Immunofluorescence of CADM1 mRNA and protein (j), CADM2 mRNA and protein (k) and RNAscope multichannel for *CADM1* (magenta) and *CADM2* (green, l). Ant, anterior; ChP, choroid plexus; Crx, neocortex; LGE, lateral ganglionic eminence; MZ, marginal zone; Pos, posterior; SVZ, subventricular zone; VZ, ventricular zone. 8 PCW scale bar = 200 μm; 10 PCW scale bar = 500 μm.

By 10 PCW, CADM2, but not CADM1, immunoreactivity showed low levels of expression in the GAP43+ growing axons of the cortical IZ and the internal capsule (IC; Figure 2h). CADM1 was instead localised to discrete cell bodies, possibly the nucleus only, in between axon bundles (Figure 2i). Within the CP and VZ, there was colocalization of *CADM1*/CADM1, *CADM2*/CADM2 and *CADM1/CADM2*. Within cell bodies, it was also apparent that *CADM1* mRNA was more highly expressed compared to protein in the SVZ, while the opposite was true of CADM2 (Figure 2j–l).

At 14 PCW, CADM2 immunoreactivity was found to colocalize with CADM1 immunoreactivity within cell bodies of the CP, but only CADM2 was strongly expressed in axons (Figure 3a,b). By 16 PCW, in a section from parietal cortex, ISH revealed a distinct band of strong *CADM2* expression within the CP but with *CADM1* expression extending across the full width of the CP. This is likely a result of differences in cortical layer expression, with *CADM2* expression strongest in layer 5 (Figure 3c). This is partially supported by our transcriptomic analysis (Figure 1d,e) in which *CADM2* was only weakly expressed in putative layer 4 cells in both prefrontal and visual cortex. The SP, outer SVZ (oSVZ) and inner fibre layer (IFL) also contained both *CADM1* and *CADM2*, with most cell bodies present expressing both mRNA species, although *CADM1* mRNA was more highly expressed than *CADM2* (Figure 3c'). *CADM1* and *CADM2* colocalization was also found within the VZ. CADM2 protein expression was found within the CP, VZ and axons of the IZ/oSVZ/IFL at 17 PCW. Here, it appeared that CADM2 was more highly expressed in axons of the IZ/oSVZ than the axons of the oSVZ/IFL (Figure 3d). This indicates CADM2 was expressed more highly in thalamocortical axons (TCA) than corticothalamic axons (CTA), as prior studies have established that TCA sit above the CTA within the cortical wall in both rodent (Deck et al., 2013; Grant et al., 2012) and primate (Alzu'bi et al., 2019) during the earliest stages of TCA ingrowth and CTA outgrowth. In addition, we found CADM2 to be weakly expressed in cortical layer 6 (Figure 3c), the origin of corticothalamic projections. CADM1 and CADM2 were colocalized in the CP and were both found within the IZ/oSVZ/IFL, where CADM1 was restricted to cell bodies surrounded by CADM2+ axon fibres, with more diffuse staining for CADM2 observed in the iSVZ (Figure 3e). Individually, both CADM1 and CADM2 colocalized with their respective mRNA in the CP. In the progenitor zones, CADM1 protein‐mRNA colocalization was found in the VZ, although only *CADM2* mRNA was present in the VZ. CADM1 and CADM2 protein expression was predominant in the iSVZ and oSVZ (Figure 3f,g).

**FIGURE 3:**
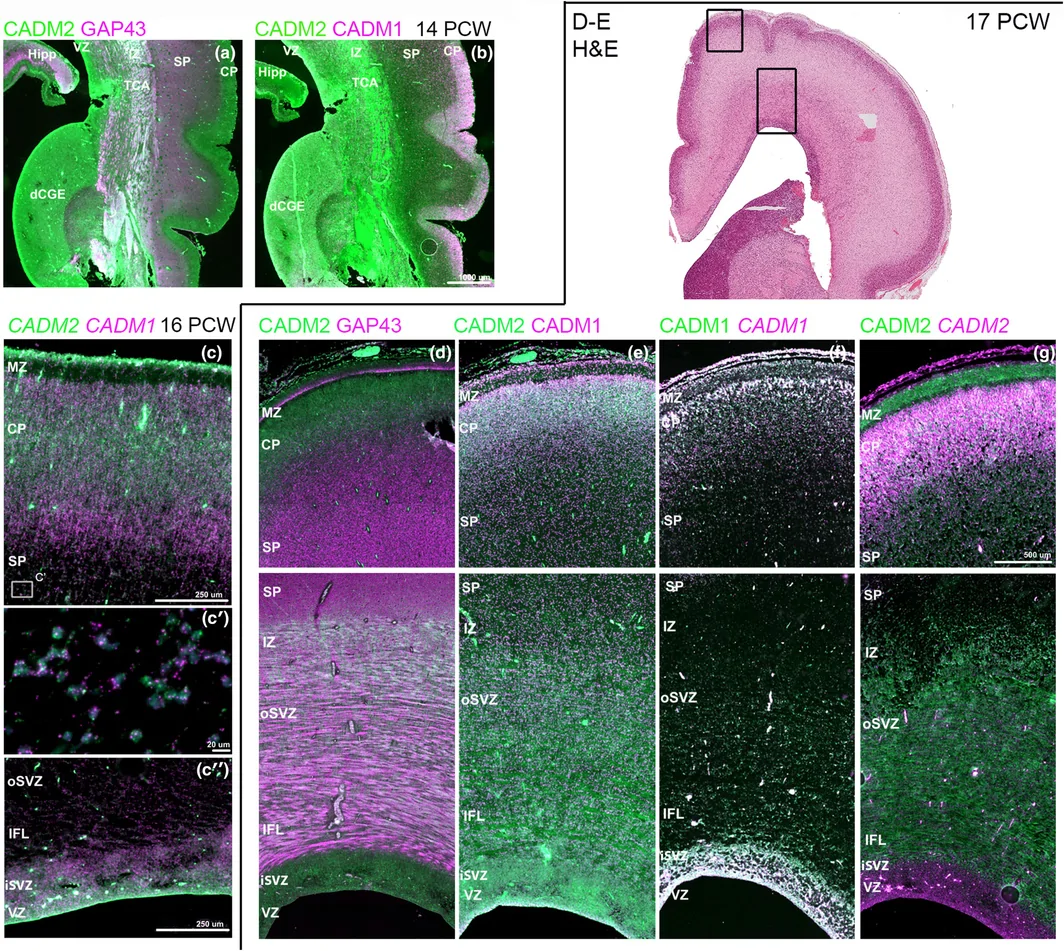
Cortical expression of CADM1 and CADM2 between 14 and 17 PCW. Fourteen PCW: Dual Immunofluorescence of the cortex stained for CADM2 (green) and GAP43 (magenta, a), and CADM2 (green) and CADM1 (magenta, b). Note colocalization of CADM2 but not CADM1 with GAP43+ thalamocortical axons (TCA; white) of the intermediate zone (IZ). Sixteen PCW: RNAscope multichannel ISH for the cortex stained for CADM1 (magenta) and CADM2 (green) mRNA in cortical plate (CP) and subplate (SP; c), cell‐level *CADM1* and *CADM2* mRNA expression in SP (c'). Note that *CADM1* is expressed across the width of the CP, but the *CADM2* signal is stronger in putative layer 5. At higher power, both mRNAs are detected in most cells in SP and CP, although CADM1 appears more highly expressed. c" shows *CADM1* and *CADM2* mRNA in the outer subventricular zone (oSVZ), inner fibre layer (IFL), inner subventricular zone (iSVZ) and ventricular zone (VZ) from the same region as c. Seventeen PCW: Lower power inset from section stained with H&E with boxes showing approximate location of higher power images d–g. Dual immunofluorescence in the cortex for CADM2 (green) and GAP43 (magenta, d) CADM2 colocalizes most strongly with GAP43 (white) in the IZ; CADM2 (green) and CADM1 (magenta, e); *CADM1* mRNA and CADM1 protein (f); *CADM2* mRNA and CADM2 protein (g). Again, CADM2 localises most strongly to the IZ. Hipp, Hippocampus; CTA, corticothalamic axons, dCGE, dorsal caudal ganglion eminence, 14 PCW scale bar = 1000 μm; 16 PCW scale bar = 500 μm C, C″, 20 μm C′; 17 PCW scale bar = 500 μm.

The cellular localization of CADM1 and CADM2 changed with the stage of development. At eight PCW, both proteins appeared to be mostly in the cytoplasm or cell membranes and in processes in the marginal zone (Figure 4a,a',a") with little staining of the cell nuclei. In the cortical plate, most cells expressed both proteins; however, in the subventricular zone, CADM1 was predominantly expressed (Figure 4a). As development progressed, CADM1 became confined to the nucleus (Figure 4b,b"); however, CADM2 continued to be expressed in the cytoplasm, at the cell surface and also in the nucleus (Figure 4b',b") as well as increasingly in axons as described above. This pattern of localization was also observed in subpallial and diencephalic regions.

**FIGURE 4:**
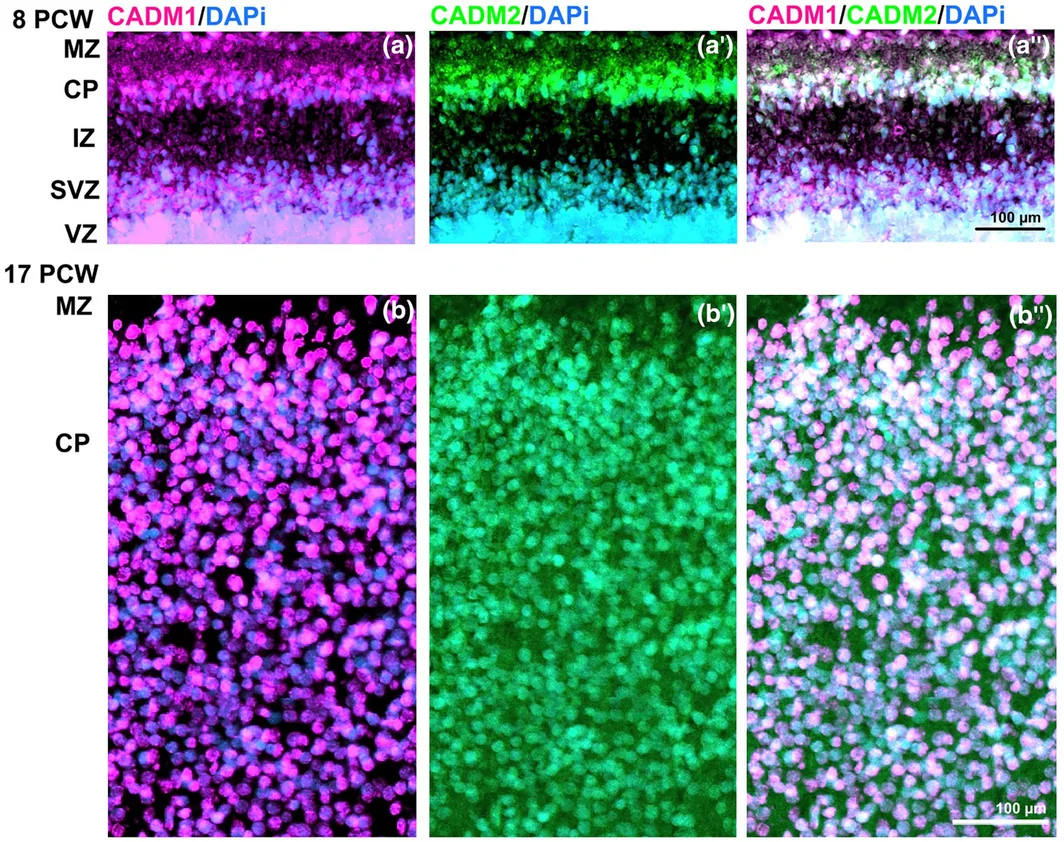
Subcellular localisation of CADM1 and CADM2. (a–a’’) CADM1 immunofluorescence (magenta) CADM2 immunofluorescence (green) and DAPi nuclear staining (blue) of the cortical wall at 8 PCW demonstrating that both proteins were predominantly localised to the cytoplasm/cell surface with limited expression in the nucleus. Most cells expressed both proteins in the cortical plate (CP) but CADM1 staining was predominant in the subventricular zone (SVZ). (b–b’’) CADM1 was almost entirely confined to the nucleus, whereas CADM2 exhibited some weak nuclear staining alongside filamentous staining that filled the spaces between the tightly packed nuclei. IZ, intermediate zone; MZ, marginal zone; VZ, ventricular zone. Scale bars = 100 μm.

### 3.2 The corpus callosum at 17 PCW

The corpus callosum is the location where SLIT2 and ROBO1 work to guide cortical axons across the midline, with secreted SLIT2 repelling growth cones expressing ROBO1 (Andrews et al., 2006; Gavrish et al., 2024; López‐Bendito et al., 2007; Shu et al., 2003; Unni et al., 2012). SLIT2 was expressed in cells in the cortical plate of the neocortex and the indusium griseum (IG), as well as the VZ (Figure 5a), while ROBO1, the axonal receptor (Liu et al., 2016), was localised to the axon containing IZ and corpus callosum (CC; Figure 5b) as expected. The CC was found to be negative for CADM1 (Figure 5c) but positive for CADM2, which colocalised with GAP43, indicating axonal expression (Figure 5d). CADM2 CC expression appeared stronger in axons directly bordering the IG. CADM1 and to a lesser extent CADM2 were expressed in the CP of the neocortex and IG (Figure 5e–g); however, CADM2 was more strongly expressed in layer 5 (Figure 5c), which would make a large contribution to transcallosal projections at this developmental stage, as layer 3 is not yet formed. The similarities between CADM1 and SLIT2 expression and between ROBO1 and CADM2 expression suggest that soluble CADM1 may act like a secreted signalling molecule (Hagiyama et al., 2009) with CADM2 as its receptor. CADM1 shows stronger mRNA than protein expression in the VZ, whereas CADM2 shows weak expression in this compartment.

**FIGURE 5:**
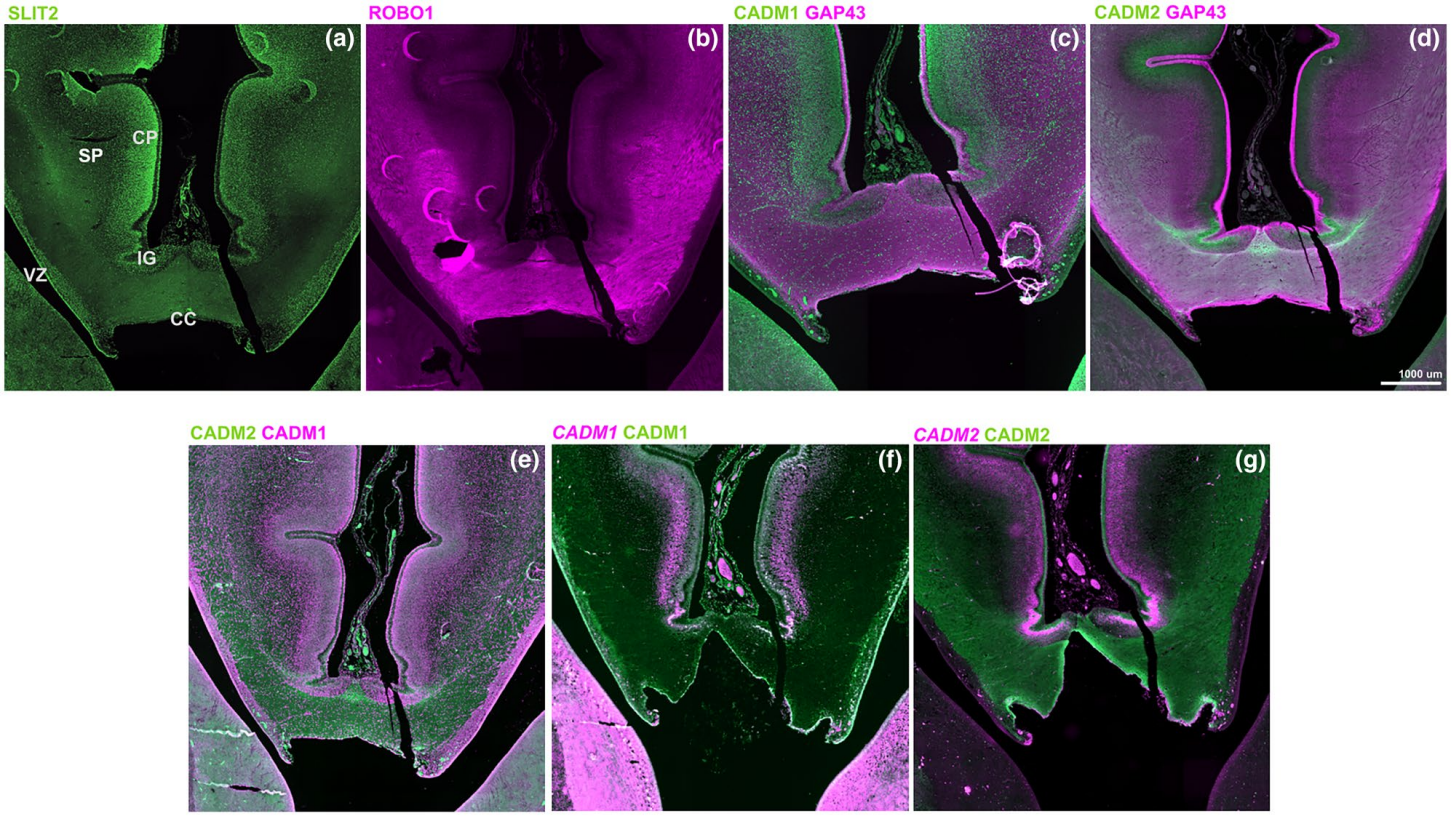
Midline expression at 17 PCW. Lower‐power inset from section stained with H&E, with box showing the approximate location of higher‐power images a–g. Immunofluorescence for SLIT2, principally located to cell bodies in the cortical plate (CP) and ventricular zone (VZ) (a) and ROBO1 localised to axons in the corpus callosum (CC) and intermediate zone (IZ) (b). Dual Immunofluorescence for CADM1/GAP43 (c), CADM2/GAP43 (d) and CADM1/CADM2 (e). RNAscope ISH + immunofluorescence for *CADM1*/CADM1 (f) and *CADM2*/CADM2 RNA (g). Note that CADM2 is predominantly localised to axon pathways and especially in axons close to the border with the indusium griseum (IG), whereas CADM1 expression is strongest in the CP and VZ. BG, basal ganglia; Crx, cortex; Dor, dorsal; IC, internal capsule; Lat, lateral; Med, medial; Ven, ventral. Scale Bar = 1000 μm.

### 3.3 Expression of CADM1 and CADM2 in the thalamus between and 17 PCW

Tissue RNA‐seq revealed that *CADM1* expression was significantly higher than *CADM2* between 8 and 17 PCW and *CADM1* tended to increase in expression between 8 and 13 PCW, whereas CADM2 expression increased slowly, if at all (Figure 6a). Single‐cell transcriptomics at 16 PCW showed similar patterns of expression to the cerebral cortex; broad expression across different cell types but with *CADM1* slightly favouring GABAergic neurons and *CADM2* favouring glutamatergic neurons and OPCs (Figure 6b). This adds extra weight to the observation that glutamatergic thalamocortical axons may strongly express CADM2 by 15 PCW onwards.

**FIGURE 6:**
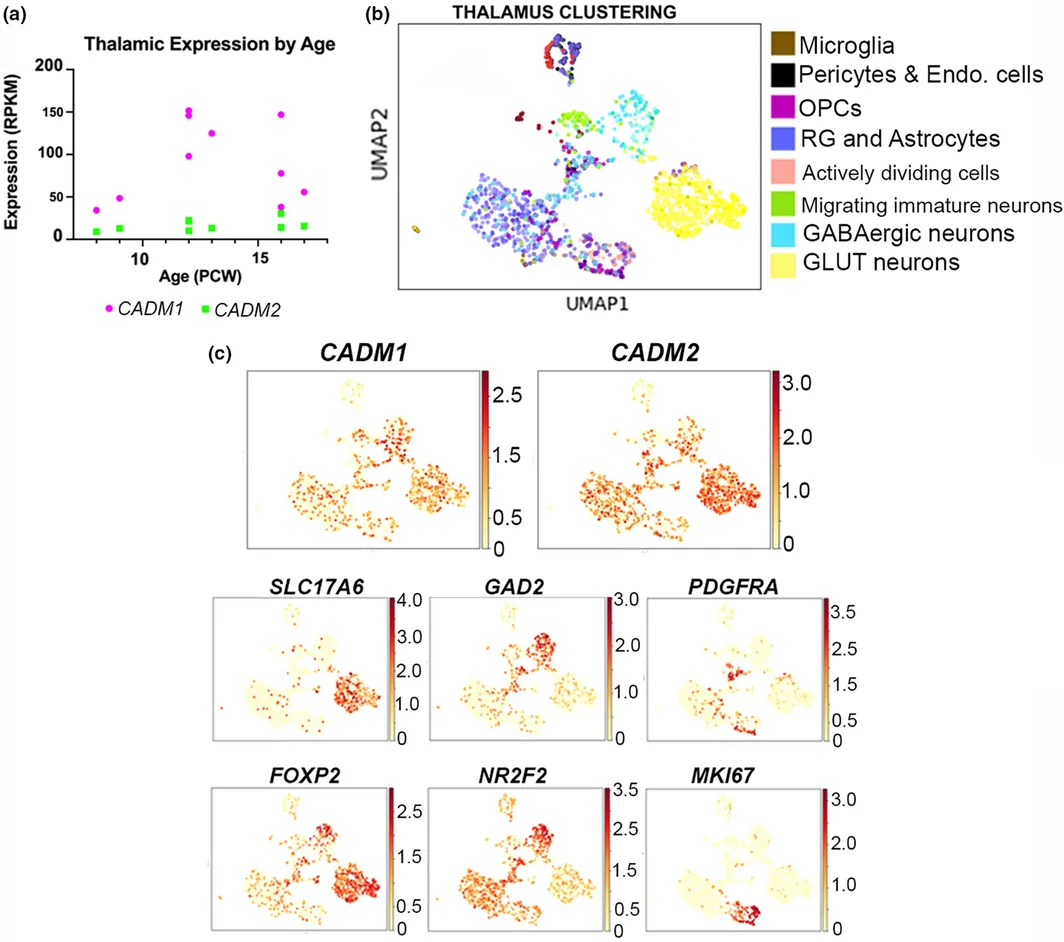
Thalamic expression of CADM1 and CADM2 between 8 and 14 PCW. Eight PCW, sagittal sections: Double immunofluorescence for CADM1/GAP43 (a), CADM2/GAP43 (b), CADM1/PAX6 (c) and CADM2/CADM1 (d). Note the strong expression of CADM2 in the PAX6+ ventricular zone of the thalamus and lack of co‐expression with the axonal marker GAP43. Ten PCW, coronal sections: Double immunofluorescence for NR2F2/FOXP2 to delineate boundaries in diencephalon (Alhesain et al., 2025); FOXP2 marks the thalamus (TH) and subthalamic nucleus (STN) and NR2F2 marks the prethalamus (pTH; e), CADM2/GAP43 (f) and CADM2/CADM1 (g) RNAScope ISH + immunofluorescence for *CADM1*/CADM1 (h) and *CADM2*/CADM2 RNA (i). Fifteen PCW: Section in the anterior/dorsal to posterior/ventral plane (see j). H&E section labelled with major thalamic nuclei at this level (k). RNAscope + immunofluorescence for *CADM1*/CADM1 (l) and *CADM2*/CADM2 (m). Note CADM1 expression confined to anterior and medial thalamic nuclei, CADM2 to posterior and lateral. Ha, Habenula; V3, third ventricle; An, anterior thalamus; MD, mediodorsal thalamus; LD, laterodorsal thalamus; pF—perifascicular complex; CM, centromedian nucleus; LGN, lateral geniculate nucleus. 8 and 10 PCW scale bar = 1000 μm, 14 PCW scale bar = 500 μm.

Immunofluorescence studies revealed that CADM1 and CADM2 proteins were both expressed in the thalamus at eight PCW. Neither colocalized with GAP43, indicating a lack of axonal expression (Figure 7a,b). CADM1 was co‐expressed with progenitor marker PAX6 within the VZ of the third ventricle (Figure 7c). CADM1 was predominantly expressed close to the ventricle, with CADM1/CADM2 colocalization in the progenitor zone of the third ventricle but also in post‐mitotic regions away from the ventricle. CADM2 immunoreactivity, but not CADM1, was observed in the prethalamus (precursor to the thalamic reticular formation; Figure 7d). At 10 PCW, in coronal sections, regions of the diencephalon and posterior hypothalamus were identified by FOXP2/NR2F2 immunostaining (Figure 7e) according to Alhesain et al. (2025). CADM2 localized to ventrolateral regions of the thalamus, medial parts of the habenula, prethalamus and the dorsolateral portion of the subthalamic nucleus, with some colocalization with GAP43+ axons within both the thalamus and prethalamus (Figure 7e–g), again providing confirmation of CADM2 expression by thalamocortical axons.

**FIGURE 7:**
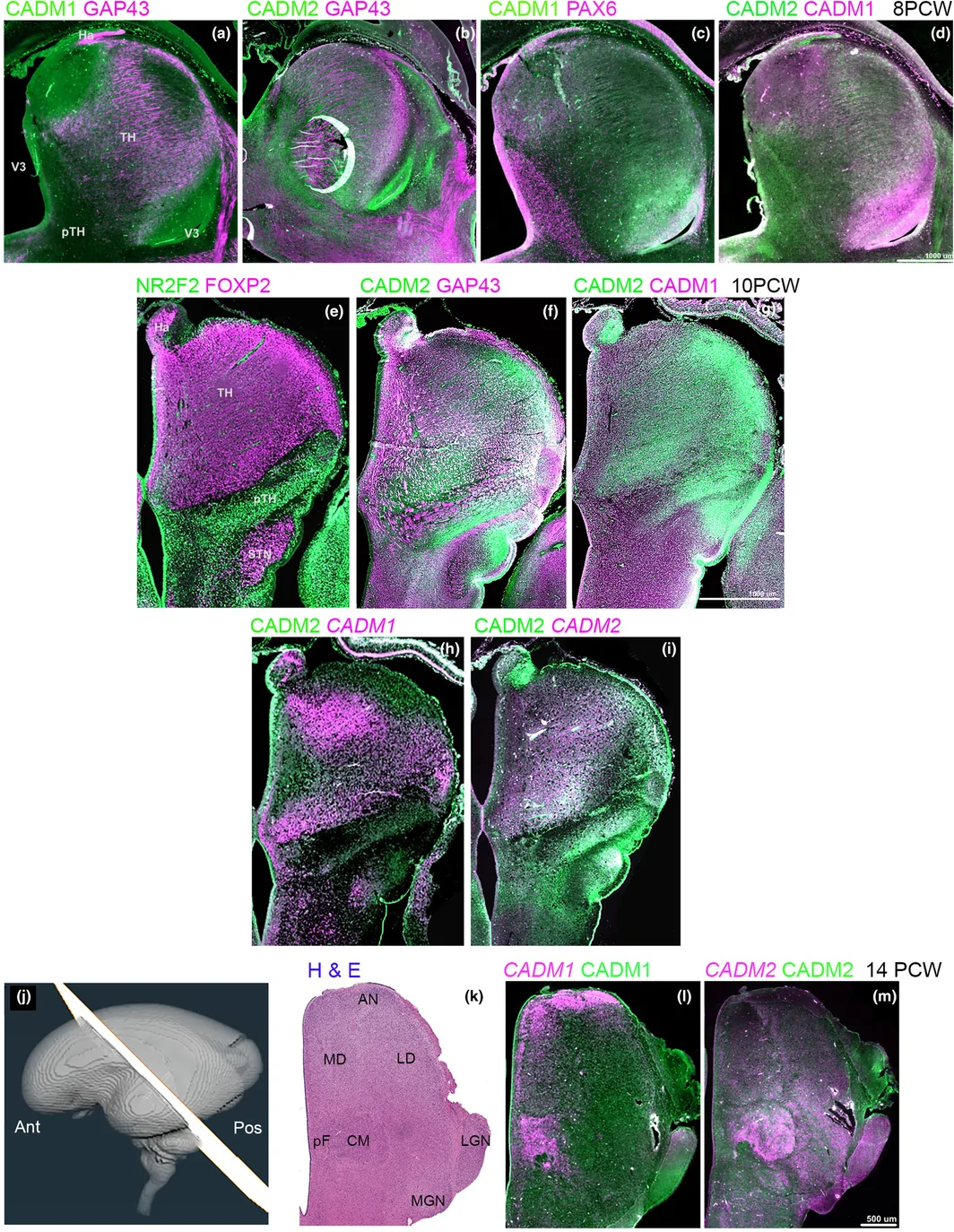
Subcellular localisation of CADM1 and CADM2. (a–a") CADM1 immunofluorescence (magenta), CADM2 immunofluorescence (green) and DAPI nuclear staining (blue) of the cortical wall at 8 PCW, demonstrating that both proteins were predominantly localised to the cytoplasm/cell surface with limited expression in the nucleus. Most cells expressed both proteins in the cortical plate (CP), but CADM1 staining was predominant in the subventricular zone (SVZ). (b–b") CADM1 was almost entirely confined to the nucleus, whereas CADM2 exhibited some weak nuclear staining alongside filamentous staining that filled the spaces between the tightly packed nuclei. MZ, marginal zone; IZ, intermediate zone; VZ, ventricular zone. Scale bars = 100 μm.

CADM1 exhibited weak expression across all regions of the thalamus and prethalamus. Weak CADM1 immunoreactivity was also found across all portions of the subthalamic nucleus; however, *CADM1* mRNA was only detected in the ventral portion which is associated with cognitive and limbic functions (Emmi et al., 2020). *CADM1* RNA was expressed in portions of the thalamus that mostly exhibited lower expression of CADM2 protein and mRNA. *CADM2* mRNA was detected throughout the thalamus and habenula but only colocalised with CADM2 protein in the lateral habenula (Figure 7h); it is in this same region of the habenula that *CADM1* and *CADM2* mRNA colocalize (Figure 7i). CADM1 and CADM2 protein are still expressed at 14 PCW throughout the thalamus (Figure 7k). However, *CADM1* mRNA was strongly expressed only in the anterior thalamus, mediodorsal nucleus and parafascicular complex. *CADM2* mRNA expression was found to be the opposite, weaker in the anterior and mediodorsal nuclei and parafascicular complex, with stronger expression in the laterodorsal, centromedian and lateral geniculate nuclei (Figure 7l).

## 4 DISCUSSION

We confirmed with transcriptomic data, RNAScope in situ hybridisation and immunohistochemistry that CADM1 and CADM2 are highly expressed throughout early fetal human forebrain development. Both glycoproteins were expressed in the cytoplasm and neurites at 8 PCW, but progressively, expression of CADM1 protein was confined to the nucleus, whereas CADM2 remained cytoplasmic and potentially on the cell surface. There was a change in the localization of CADM2 immunoreactivity from no axonal expression at 8 PCW to axonal expression at 10 PCW and a strengthening of expression in certain axon tracts as development progressed, indicating a role in axon fasciculation and possibly guidance.

### 4.1 CADM1 expression

Surprisingly, as CADM1 is generally regarded as a membrane‐bound cell‐adhesion molecule, from 10 PCW onwards we observed weak expression of CADM1 in the cytoplasm, no expression in axons and strong expression within the cell nucleus within the fetal forebrain. These observations were contradictory to some established cancer studies which have demonstrated the localisation of CADM1 to the cell membrane (Fujita et al., 2010; Fujita et al., 2012; Hagiyama et al., 2009). As paraformaldehyde fixation and delipidation might permeabilise and disrupt cell membranes, potentially reducing the number of molecules localised to the cell membrane while enhancing intracellular detection, it is possible that detection of cell surface receptors is suboptimal under our experimental conditions.

However, the observed nuclear localisation of CADM1 is likely to be real as this has been observed before. For instance, in the cervical epithelium, expression is observed in the cell membrane of glandular epithelial cells but in the nucleus of squamous epithelial cells. Further investigation revealed that the complete protein is transported to the nucleus or nuclear envelope, suggesting it may have a direct role in transcriptional regulation (Overmeer et al., 2008). Other cell surface glycoproteins have been observed to demonstrate nuclear expression during brain development, notably neurexins 1 and 3 (Harkin et al., 2017), the ERBB family of tyrosine kinase receptors (with, or without, proteolytic cleavage; Carpenter, 2003) and FGFR1 (Reilly & Maher, 2001).

The observed nuclear expression of CADM1 may be due to the lack of CDH1 (E‐cadherin) expression in the developing forebrain. A change in CADM1 localisation from cell membrane to nucleus has been observed in U251 cell cultures upon the degradation of CDH1 via tissue plasminogen activator treatment (Yageta et al., 2002). According to the tissue RNAseq data interrogated in this study, *CDH1* exhibits low expression in the human fetal forebrain, with expression levels a sixtieth of those detected for *CDH2* (N‐cadherin; Lindsay et al., 2016). This aligns with other research that demonstrates weak transient expression in both human (Lewis‐Tuffin et al., 2010) and embryonic mouse brain (Karpowicz et al., 2009).

It has been observed that CADM1 serves to promote the formation of epithelial‐like structures when over‐expressed in HEK cells, binding cells together in a structured tissue (Sakurai‐Yageta et al., 2009). This explains why reduced expression of CADM1 in cancerous cells increases cell motility. The cytoplasmic/cell surface expression of CADM1 seen at 8 PCW, when the cortical plate is first forming, may play a role in allowing a coherent layer of CP cells to aggregate and effectively split the preplate. Downregulation of CADM1 in cultured human mast cells results in reduced adhesion to other cells and the extracellular matrix, which occurs both directly through CADM1‐dependent adhesion and indirectly through the regulation of other adhesion receptors (Moiseeva et al., 2014). As development progresses and the distances from the ventricular zone to the cortical plate increase, lack of membrane‐expressed CADM1 may serve to facilitate post‐mitotic neuron migration to their final destinations. At the same time, increased expression in the nucleus may suppress neurogenesis and promote differentiation to a neuronal phenotype. Nuclear localisation of retinoblastoma (RB) protein is upregulated in the presence of CADM1, which in turn leads to the arrest of cell division (Zhang et al., 2016).

Alternatively, or in addition, CADM1 is known to undergo ectodomain shedding, in which the activity of the secretase ADAM10 releases the extracellular portion of the molecule (sCADM1; Shirakabe et al. 2017), which is the part recognised by the antibody used in this study, into the extracellular space where presumably it can act as a soluble ligand. If significant amounts of shedding occur, this might reduce the concentration at the cell membrane. sCADM1 can then interact with CADM2 or other molecules expressed on the cell surface of developing neurons and their neurites. It has been demonstrated that sCADM1 attracts neurite outgrowth by binding membrane‐bound CADM1 (Hagiyama et al., 2009).

### 4.2 CADM2 expression and interaction with CADM1 at the midline

CADM2 is involved in maintenance of cell polarity and adhesion by interacting with the extracellular matrix and, like CADM1, suppresses tumour growth in different types of cancers (Chang et al., 2010; He et al., 2013). However, in metastasising cancer cells in the brain, CADM2 appears to promote a migratory cell phenotype (Dai et al., 2020). Therefore, in the context of the developing brain, CADM2 expression in the leading neurite of either a migrating cell or growing axon may be involved in guiding migration of cells or axon extension, by interaction with CADM1 or other binding partners.

We have demonstrated increasing expression of CADM2 in axons during development, especially in thalamocortical axons and corticocortical axons as they cross the corpus callosum, although corticofugal axons appear to show lower levels of expression by 17 PCW (Figures 3 and 7). It is possible that CADM2‐CADM2 interactions are involved in fasciculating axons within certain tracts, keeping them on their path towards their target. As CADM2 immunoreactivity is greater in the IZ than in the SP, it would appear that CADM2 expression is downregulated in thalamocortical axons branching off from the main tract and invading the subplate. Therefore, although CADM2 is regarded as important in synapse formation and maintenance, it is not demonstrated here to be involved in early cortical synaptogenesis in the subplate.

In the corpus callosum, CADM2 expression and colocalization with GAP43 is greatest at the dorsal boundary with the indusium griseum. Whereas SLIT2 secreted from cortical plate cells may serve to repel ROBO1+ cortico‐cortical axons into the corpus callosum and away from the CP, release of soluble CADM1 from the CP may serve to pull CADM2 expressing axons across the corpus callosum. Working together, SLIT2/ROBO1 repulsion and CADM1/CADM2 attraction may ensure axons cross the midline and grow onwards without recrossing. CADM2 and CADM1 have been demonstrated to be involved in commissural axon growth across the spinal cord in concert with ROBOs and SLITs, although CADM2 is expressed by glia floorplate cells and both CADMs are expressed on growth cones, so the mechanisms involved would not be the same (Frei et al., 2014; Niederkofler et al., 2010). Defects in corpus callosum formation, both hypoplasia and slower growth, have been implicated in neurodevelopmental diseases (Gilliam et al., 2011; von Plessen et al., 2002; Wolff et al., 2015), and this might help explain how CADMs can be susceptibility genes for a variety of conditions.

Despite the changing expression of CADM2 its colocalization with CADM1 within the cortical plate continued to be observed across ages. At 16 PCW, *CADM1* and *CADM2* mRNA were observed colocalizing to the same cells within the CP and SP. In mice, same cell localization of CADM1 and CADM2 has been found to be critical to their cis‐interaction with the H protein, which allows for virus‐cell membrane fusion at the synapse (Shirogane et al., 2021). Both cis and trans‐interactions of CADMs at the pre‐ and post‐synaptic membranes have been shown to be important in inducing and maintaining synapse formation in experimental models (Biederer et al., 2002; Fogel et al., 2007; Frei & Stoeckli, 2014). Therefore, it was surprising to see very little expression of either CADM in neurites in the SP which, along with the marginal zone, is the earliest site of synapse formation in the developing cortex (Kostović, 2024). It may be that CADMs act principally in guiding migration of cells and growth and fasciculation of axons during early human development.

### 4.3 Roles for CADM1 and CADM2 in the thalamus and associated structures

Transcriptomic data, in situ hybridisation and immunofluorescence all confirmed expression of CADM2 by thalamic projection neurons, supporting our contention that thalamocortical axons strongly and progressively express CADM2 throughout development. However, further examination of localisation of expression patterns revealed that, unlike in the cortex, differential expression of these molecules may be involved in the emerging arealisation of the dorsal thalamus and associated structures into functional nuclei, which occurs by 14 PCW in the human thalamus (Alhesain et al., 2025). CADM2 showed preferential expression in lateral and ventral regions of the thalamus at 10 PCW that became more distinct by 14 PCW, whereas CADM1 expression became largely confined to medial and anterior nuclei. The preferential expression of CADM1 in the parafascicular complex and CADM2 in the centromedian nucleus was unexpected as these nuclei are functionally similar and have been shown to share developmental markers in a previous study in humans (Alhesain et al., 2025).

Similarly, the habenula was divided into medial and lateral portions by CADM2 expression, and the subthalamic nucleus into ventromedial and dorsolateral compartments. The medial habenula contains cholinergic neurons and projects exclusively to the interpeduncular nucleus in the mature brain, whereas the lateral habenula projects to multiple targets in the midbrain and hypothalamus (Ables et al., 2023; Hones & Mizumori, 2022). The CADM2 expressing ventral and lateral thalamus and dorsolateral subthalamic nucleus are associated with somatosensory and sensorimotor functions in the mature brain (Lenz et al., 1999; Marcuse et al., 2025; Mosher et al., 2021), whereas CADM1 expressing medial thalamic nuclei and ventromedial subthalamus nucleus are more associated with cognitive and limbic functions (Emmi et al., 2020; Nelson, 2021; Pergola et al., 2018).

Thus, it appears that differential expression of CADMs may help specify the functional arealisation of the diencephalon. This may involve guiding axon growth towards the correct targets, leading to further phenotypic refinement guided by retrograde signalling or, perhaps, ensure cells of the same phenotype aggregate within the same location, as has been described for other cell surface receptors (Cooke & Moens, 2002).

## 5 CONCLUSIONS

In the cortex, CADM1 and CADM2 localise to the same cells, although CADM2 favours the cell surface and neurites while CADM1 favours the cytoplasm and nucleus. CADM2 appears to play a role in axon fasciculation and directional growth of subsets of axons. Nuclear CADM1 might play a role in regulating neurogenesis, whereas release of sCADM1 might act to attract migrating cells or growth cones that express CADM2. Such hypotheses remain to be tested in future studies. In the thalamus and associated structures, differential expression of CADMs appears to play a role in the regionalisation of the thalamus into distinct functional nuclei. Therefore, CADM1 and CADM2 are likely to be involved in several early developmental processes that, if perturbed, could lead to altered cortical circuit connectivity.

## Data Availability

The data that support the findings of this study are available from the corresponding author upon reasonable request.
